# Glycogen storage disease type III presenting with secondary diabetes and managed with insulin: a case report

**DOI:** 10.4076/1757-1626-2-6891

**Published:** 2009-06-17

**Authors:** Heba Ismail

**Affiliations:** 1 Diabetes, Endocrinology and Metabolism Pediatric Unit, University of Cairo, Egypt

## Abstract

**Introduction:**

Reports of secondary diabetes in glycogen storage disease type III have been very limited, where the pathogenesis and management have not been clear. Here we report on a rare case of secondary diabetes in glycogen storage disease type III that has been successfully managed with insulin.

**Case presentation:**

This is a 19-year-old female of Egyptian ethnicity, born of a consanguineous marriage and known to have glycogen storage disease type III since the age of 2½ years. She presented to us with a history of polyuria, polydipsia, and loss of weight of a few days duration. Physical exam showed stunted growth, hepatomegaly, myopathy and mild dehydration. Emergency labs revealed a fasting blood glucose of 276 mg/dl, but with no ketonuria and arterial blood gases were essentially normal. Her liver transaminases were mildly elevated at the time. Review of her records revealed that the diagnosis of glycogen storage disease type III was made at the age of 2½ when the mother reported repeated attacks of afebrile (hypoglycemic) convulsions, increasing abdominal girth and failure to thrive. The diagnosis was confirmed by demonstration of debrancher enzyme deficiency on enzymatic assay. Over the years she developed liver dysfunction along with other complications and subsequently her hypoglycemic attacks disappeared a few years prior to her current presentation. After careful consideration of different treatment options, and considering she had been free of hypoglycemic attacks for a few years and had liver dysfunction, we chose to cautiously initiate the patient on insulin therapy. She was still poorly controlled and we gradually increased her total daily dose to 0.8 u/kg. She continued to be free of hypoglycemic attacks and her average daily blood glucose is about 160 mg/dl.

**Conclusion:**

We report a rare case of secondary diabetes mellitus in a patient with glycogen storage disease type III managed with insulin. We recommend insulin therapy over oral hypoglycemics to avoid further hepatotoxicity, provided hypoglycemia has resolved. We also recommend serial follow up of glycogen storage disease type III patients with an oral glucose tolerance test for early detection and management of glucose intolerance.

## Introduction

Glycogen-storage disease type III (GSD III) is an autosomal recessive metabolic disorder caused by loss of function mutations of the glycogen debranching enzyme (Amylo-1,6-glucosidase) gene, located at chromosome band 1p21.2. It is characterized by the storage of abnormal glycogen in both skeletal and cardiac muscle and/or the liver, with variability in resultant organ dysfunction. Major manifestations of GSDIII include hypoglycemia (which may improve and disappear with age), hepatomegaly with elevated transaminases, hyperlipidemia, hyperuricemia, and skeletal myopathy/cardiomyopathy with increased creatine phosphokinase (CPK). There is no specific treatment for GSDs, but diet therapy with nocturnal nasogastric tube feeding and cornstarch improves symptoms (especially hypoglycemia), reduces the liver size, and improves overall growth and development [1]. Rare complications of GSDIII include liver cirrhosis and failure together with secondary glucose intolerance and frank diabetes. However, reports of such cases have been very few. Diabetes mellitus (DM) in GSD III is difficult to treat because patients are prone to hypoglycemia. We, however, report on a case of GSDIII with secondary diabetes successfully controlled with insulin.

## Case presentation

This is a 19-year-old female of Egyptian ethnicity, born of a consanguineous marriage and known to have glycogen storage disease type III since the age of 2½ years. She presented to us with a history of several days of polyuria, polydipsia, and loss of weight.

Review of her records revealed that she had been diagnosed with GSDIII at the age of 2½ years, when the mother reported an increasing abdominal girth, failure to thrive, and repeated attacks of afebrile convulsions of 1 year duration. The diagnosis of GSDIII was confirmed at the time by a liver biopsy showing a picture suggestive of GSD (swollen cells and mosaic cytoplasmic appearance with mild fibrosis) as well as demonstration of debrancher enzyme deficiency. She was prescribed long-term therapy with cornstarch for hypoglycemia and colchicine for associated hyperuricemia. Unfortunately, there was variable follow-up by the mother together with non-adherence to management regimens.

Over the years she developed progressive chronic liver dysfunction with persistently high liver transaminases and γ-glutamyl transpeptidase together with coagulation profile compromise and bleeding tendency. Jaundice was documented only once during an attack of acute hepatitis on top of her chronic dysfunction. Her most recent follow-up liver biopsy (done years prior to her current presentation) had shown increased fibrosis and changes suggestive of very early cirrhosis, but with intact limiting plates. Subsequently, evidence of vitamin D and A deficiencies developed with metastatic soft tissue calcifications and osteoporotic bone fractures, together with poor night vision and dry skin, respectively. Moreover, the patient later developed myopathy, with progressive muscle weakness and pain, indicating a diagnosis of GSDIII type a (both liver and muscle involvement). Fortunately, about four years prior to her current presentation the patient's hypoglycemic attacks improved and eventually disappeared. Her family history is positive for two younger sibs, both diagnosed with GSD.

Upon physical exam, she appeared dysmorphic, stunted, with a mild degree of dehydration and dry skin. Abdominal exam revealed a firm and enlarged liver, but with no splenomegaly or ascites. Neuromuscular system examination revealed reduced muscle power in both upper and lower limbs, while cardiovascular system exam excluded any evidence of cardiomyopathy. Pubertal staging revealed primary amenorrhea, but with adequate development of secondary sex characters. Positive clinical findings are summarized in Table 1.

**Table 1:** Clinical presentation of our case

Physical Examination	Positive Findings
General appearance	Facial dysmorphism and stunted growth
Anthropometry	Height -2.2 SDS, Weight -4 SDS
Skin	Dry skin and mild dehydration
Abdominal	Liver ≈ 5 cm below the costal margin and firm
Neuromuscular	Bilateral and symmetrically reduced muscle power (4/5) in both upper and lower limbs

Emergency labs done at the time of presentation revealed a fasting blood glucose (FBG) of 276 mg/dl and no ketones. Her arterial blood gases (ABG) were essentially normal with a pH of 7.36, PCO_2_ 44.1, HCO_3_ 22.1, BE -1.1.

Further lab studies showed pus cells in urine, and a culture later grew *Escherichia coli.* A complete blood count with a differential count showed a marked shift to the left. Her liver transaminases, γ-glutamyl transpeptidase and alkaline phosphatase were mildly elevated, while her kidney functions and electrolytes were normal. Her uric acid and lactate levels were also normal, but her creatine phosphokinase was high at the time.

**Figure 1:**
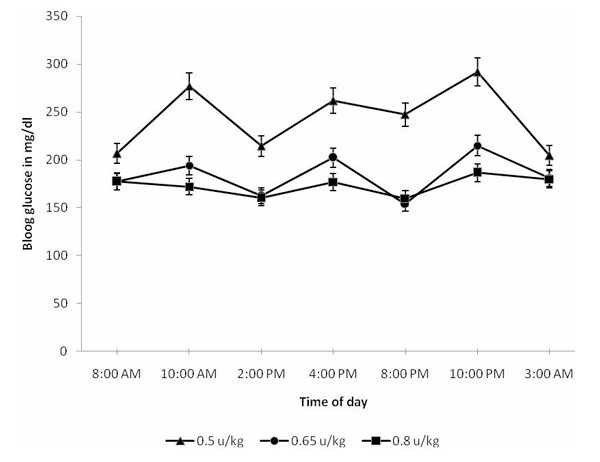
**Daily blood glucose levels on different insulin doses**.

After careful consideration of different treatment options and considering the patient was free of hypoglycemic attacks for an adequate time without treatment and that she had already developed liver dysfunction and early cirrhosis, we decided to begin the patient on very low doses of insulin therapy. She continued to show high blood glucose (BG) readings with a daily average of about 263 mg/dl, requiring that we gradually increase her total dose to about 0.8 u/kg/day. She is currently relatively well controlled and has continued to be free of hypoglycemic attacks; rather she tends to err on the high side with a daily average BG of approximately 160 mg/dl. Figure 1 shows average daily glucose levels during the initial period of dose adjustment.

## Discussion

Reports on the pathogenesis of secondary diabetes in GSDIII and its management have been very limited and relatively unclear [2,3]. Glucose intolerance and secondary diabetes in GSDIII is thought to result from liver dysfunction. The pathogenesis of glucose intolerance in liver dysfunction in general also remains unclear, where several factors are thought to be responsible, including insulin resistance, insulin hypersecretion with reduced clearance, impaired glycogen catabolism, defective adipocyte insulin sensitivity and others. Diabetes development in patients with liver cirrhosis, regardless of etiology, shows that about 80% develop glucose intolerance, while up to 40% can develop frank DM [4,5]. Early on there is post-prandial hyperglycemia (PPHG), but later on there is both fasting & PPHG. Of note here is that our patient had already developed liver dysfunction and early cirrhosis at the time she presented to us. She was unfortunately missed during the earlier stages of intolerance and PPHG due to her loss to follow up.

Classically, DM associated with liver cirrhosis is treated with insulin; however, this becomes a major problem if cirrhosis is secondary to GSDIII due to the high risk of hypoglycemia. Oki et al. [3] described an adult GSDIII patient who later developed liver dysfunction. This dysfunction worsened over the years, and he subsequently developed PPHG. Being that this patient presented with PPHG, the authors chose to treat him with an α-glucosidase inhibitor, voglibose. Alpha-glucosidase inhibitors blunt PPHG by essentially delaying the hydrolysis of carbohydrates in the intestines. Accordingly, there is a low risk for hypoglycemia. However, when this option was considered in our patient, it was thought unhelpful given that she had presented to us at a more advanced stage of fasting hyperglycemia. Furthermore, there has been conflicting evidence regarding the potential hepatotoxicity of voglibose [6]-[8]. So considering our case had elevated liver transaminases, we chose not to take this risk.

Due to the patient's presentation with fasting hyperglycemia, the spontaneous resolution of her hypoglycemic attacks for several years prior to clinical diabetes, and the potential hepatotoxic effect of most oral hypoglycemics, we decided to manage her diabetes with insulin therapy and have been so far successful.

## Conclusion

We report a rare case of secondary DM in a GSDIII patient who has been successfully managed with insulin. We recommend insulin therapy over oral hypoglycemics to avoid further hepatotoxicity, provided hypoglycemias have resolved. We also recommend serial follow up of GSDIII patients with an oral glucose tolerance test (OGTT) for early detection and management of glucose intolerance.

## Abbreviations

BG: Blood glucose; DM: Diabetes Mellitus; GSD: Glycogen storage disease; GSDIII: Glycogen storage disease type III; OGTT: Oral glucose tolerance test; PPHG: Post-prandial hyperglycemia; SDS: Standard deviation score.

## Consent

Written informed consent was obtained from the patient's mother for publication of this case report. A copy of the written consent is available for review by the Editor-in-Chief of this journal.

## Competing interests

The author declares that she has no competing interests.

## Author contribution

HI provided initial management and follow up of this case's diabetes.
